# A Fatal Case of Rapidly Progressive Glomerulonephritis With Two Anti-neutrophil Cytoplasmic Antibodies and Anti-glomerular Basement Membrane Antibody: A Description of Autopsy Findings

**DOI:** 10.7759/cureus.44160

**Published:** 2023-08-26

**Authors:** Hanako Ishikawa, Yuki Ota, Keisuke Iwasaki, Kumiko Muta, Tomoya Nishino

**Affiliations:** 1 Anesthesiology, Nagasaki University Graduate School of Biomedical Sciences, Nagasaki, JPN; 2 Nephrology, Sasebo City General Hospital, Sasebo, JPN; 3 Pathology, Sasebo City General Hospital, Sasebo, JPN; 4 Nephrology, Nagasaki University Hospital, Nagasaki, JPN

**Keywords:** autopsy, hemodialysis, anti-glomerular basement membrane disease, anca associated vasculitis, acute kidney injury, rapidly progressive glomerulonephritis

## Abstract

A 79-year-old man presented with dyspnea upon exertion, marked renal dysfunction, proteinuria, and hematuria. He was diagnosed with rapidly progressive glomerulonephritis. Serological tests were positive for MPO-ANCA, PR3-ANCA, and anti-GBM antibodies. Since the anti-GBM antibody titer was significantly higher than the ANCA titer and the renal dysfunction was severe, we initially assumed anti-GBM disease and started treatment. Due to poor general condition, a definitive diagnosis could not be made by renal biopsy. Corticosteroid therapy, plasmapheresis, and cyclophosphamide treatment were performed. However, renal function did not improve, and hemodialysis was required. He died of sepsis during treatment. An autopsy was performed with the consent of the family. Renal pathological examination revealed fibrocellular crescent formation in the glomeruli. Immunofluorescence revealed no major deposition in the glomeruli, suggesting ANCA-associated nephritis but not anti-GBM disease. Gross pathological findings of the abdominal aorta showed that a part of the artificial blood vessel had formed a pseudoaneurysm and abscess. There is no evidence of inflammatory cell infiltration or vasculitis in the alveoli. Pathological findings in the other organs did not suggest vasculitis. The renal prognosis of this case could have been improved with appropriate treatment if early diagnosis by renal biopsy had been made. There have been case reports of triple-seropositive rapid progressive glomerulonephritis (RPGN). We report a rare autopsy case of triple-seropositive RPGN.

## Introduction

Rapid progressive glomerulonephritis (RPGN) is defined by the World Health Organization as a syndrome characterized by acute or latent-onset hematuria, proteinuria, anemia, and rapidly progressive renal failure. Crescentic glomerulonephritis conditions, such as antineutrophil cytoplasmic antibody (ANCA)-associated nephritis and anti-glomerular basement membrane (GBM) disease, are typical examples of RPGN. Single ANCA-positive and double seropositive cases are the most common; however, triple seropositive RPGN cases with myeloperoxidase-ANCA (MPO-ANCA), proteinase-3-ANCA (PR3-ANCA), and anti-glomerular basement membrane (GBM) antibodies have also been reported [1,2]. However, the number of case reports is small, and there is no consensus regarding the pathology and treatment of triple-seropositive RPGN. Here, we report a rare autopsy case of triple-seropositive RPGN. This article was previously presented as a meeting abstract at the 67th Annual Meeting of the Japanese Society for Dialysis on July 3.

## Case presentation

A 79-year-old man presented to our hospital with chief complaints of shortness of breath on exertion and leg edema. He had undergone aortic valve replacement and coronary artery bypass surgery for aortic regurgitation and angina eight years before. One year prior to presentation, he had undergone graft replacement for a thoracoabdominal aortic aneurysm. After surgery, the patient visited the outpatient department of the Cardiovascular Surgery Department. One month before presentation, he began to feel fatigued and gradually developed shortness of breath upon exertion, as well as edema in the lower extremities. Blood and urine tests revealed severe renal dysfunction, proteinuria, and hematuria, and the patient was immediately hospitalized.

The patient’s physical findings on admission were as follows: body height, 168 cm; body weight, 62.5 kg; body temperature, 36.5°C; blood pressure, 139/74 mmHg; heart rate, 75/min; and percutaneous oxygen saturation (SpO2), 97% (FiO2 25%). On auscultation, there were no abnormalities in heart and breathing sounds. Pitting edema was observed in the lower leg on palpation. No eruptions or purpura were observed on inspection. No neurological abnormalities were observed. The prescribed drugs were amlodipine, carvedilol, aspirin, lansoprazole, and sodium ferrous citrate.

A blood test revealed a worsening renal function with serum creatinine (Cr) of 7.1 mg/dL (normal range 0.65-1.07). One month ago, the Cr value was 2.1 mg/dL due to nephrosclerosis. Urinalysis revealed proteinuria with a urinary protein creatinine ratio (UPCR) of 4.21 g/gCr and hematuria with erythrocytes of 20-29/HPF. A diagnosis of rapidly progressive glomerulonephritis was made based on the clinical course. Autoantibody measurements were positive for MPO-ANCA, PR3-ANCA, and anti-GBM antibodies (MPO-ANCA, 26.9 IU/mL normal range 0-5; PR3-ANCA, 17.3 IU/mL normal range 0-3; and anti-GBM antibody, 235.4 U/mL normal range 0-7, Table 1).

**Table 1:** Laboratory Findings Alb, albumin; ALT, alanine transaminase; ALP, alkaline phosphatase; AST, aspartate aminotransferase; BUN, blood urea nitrogen; β­₂MG, beta-2 microglobulin; Cl, chloride; Cr, creatinine; CRP, C-reactive protein; eGFR, estimated glomerular filtration rate; GBM, glomerular basement membrane; γGTP, gamma-glutamyltransferase; Hb, hemoglobin; Hct, hematocrit; IgA, immunoglobulin A; IgG, immunoglobulin G; IgM, immunoglobulin M; K, potassium; LDH, lactate dehydrogenase; Na, sodium; MPO-ANCA, myeloperoxidase-anti-neutrophil cytoplasmic antibodies; NAG, N-Acetylglucosaminidase; PLT, platelet; PR3-ANCA, proteinase-3-anti-neutrophil cytoplasmic antibodies; RBC, red blood cell; TP, total protein; UPCR, urinary protein creatinine ratio; WBC, white blood cell

[Blood test] (normal range)				[Urinalysis] (normal range)	
WBC	6300/μL (3300-8600)	Na	137 mEq/L (138-145)	Protein	(2+)
RBC	289 x 10^4^ /μL (435-555 × 10^4^)	K	3.8 mEq/L (3.6-4.8)	Glucose	(-)
Hb	9.4 g/dL (13.7-16.8)	Cl	107 mEq/L (101-108)	Urobilinogen	(-)
Hct	26.8% (40.7-50.1)	Ca	8.1 mg/dL (8.8-10.1)	Bilirubin	(-)
PLT	8.3 × 10^4^ /μL (15.8-34.8 × 10^4^)	P	6.0 mg/dL (2.7-4.6)	Ketone	(-)
TP	6.2 g/dL (6.6-8.1)	CRP	1.72 mg/dL (0-0.14)	RBC	20-29/HPF
Alb	3.0 g/dL (4.1-5.1)	IgG	1276 mg/dL (801-1747)	WBC	30-49/HPF
AST	16 U/L (13-30)	IgA	398 mg/dL (93-393)	UPCR	4.21 g/gCr
ALT	10 U/L (10-42)	IgM	52 mg/dL (33-183)	NAG	36.8 U/L (0.9-6.2)
γGTP	22 U/L (13-64)	C3	76 mg/dL (73-138)	β₂MG	14179 μg/L (30-340)
LDH	185 U/L (124-222)	C4	26 mg/dL (11-31)		
UA	10.7 mg/dL (3.7-7.8)	CH50	65 U/mL (32-58)		
BUN	53.5 mg/dL (8-20)	MPO-ANCA	26.9 U/mL (0-5)		
Cr	7.16 mg/dL (0.65-1.07)	PR3-ANCA	17.3 U/mL (0-3)		
eGFR	6.4 ml/min/1.73 m^2^	Anti-GBM antibody	235.4 U/mL (0-7)		

**Figure 1:**
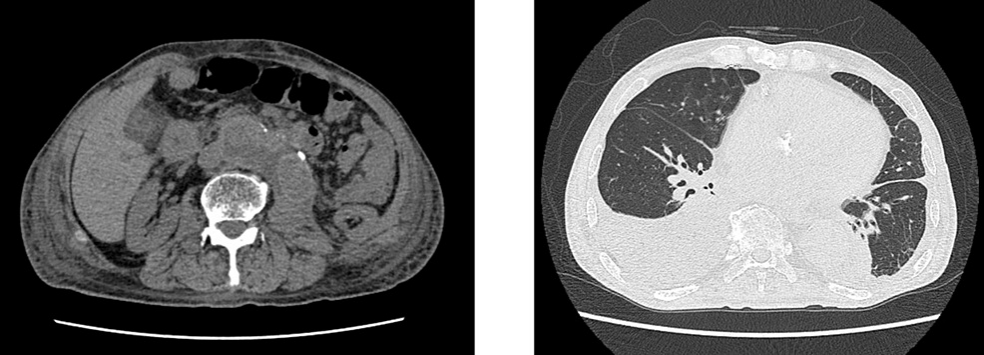
Computed tomography findings The left kidney was atrophied compared with the right kidney. Bilateral pleural effusion and right atelectasis are observed. There is no interstitial lung disease or alveolar hemorrhage. An abscess was suspected in the structure on the right side of the artificial blood vessel.

These results suggested either ANCA-associated nephritis or anti-GBM disease. Computed tomography findings showed bilateral pleural effusions and no evidence of interstitial pneumonia or alveolar hemorrhage. An abscess was suspected in the structure on the right side of the artificial blood vessel (Figure 1).

**Figure 2:**
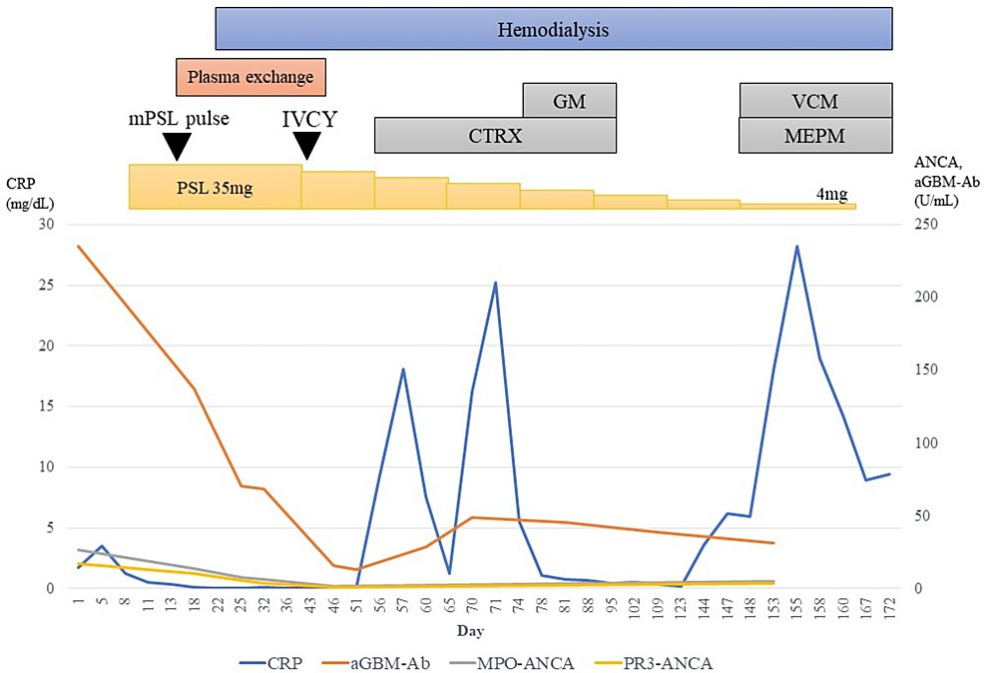
Clinical course aGBM-Ab, anti-glomerular basement membrane antibody; CTRX, ceftriaxone sodium hydrate; GM, gentamicin sulfate; MEPM, meropenem hydrate; MPO-ANCA, myeloperoxidase-anti-neutrophil cytoplasmic antibodies; mPSL, methylprednisolone; PR3-ANCA, proteinase-3-anti-neutrophil cytoplasmic antibodies; PSL, prednisolone; VCM, vancomycin hydrochloride

At first, heart failure was thought to have caused the worsening of renal function. Therefore, heart failure was treated with furosemide and tolvaptan for the first 14 days. As a result, diagnosis and treatment were delayed as the autoantibody test was not submitted. Although the patient’s ANCA titer was high, his anti-GBM antibody titer was markedly higher; therefore, it was assumed that anti-GBM disease was the primary pathology. Because one kidney was atrophied, a renal biopsy was considered high risk and was not performed. On day 14 of hospitalization, methylprednisolone was administered at 1 g/day for three days, followed by prednisolone at 35 mg/day (0.6 mg/kg/day). Plasma exchange was performed for three weeks from day 14 of hospitalization. However, renal dysfunction did not improve, urine output decreased, body weight increased, and systemic edema worsened. Thus, hemodialysis was initiated on the 35th hospital day. Intravenous cyclophosphamide was administered only once on the 43rd hospital day. His MPO-ANCA titer decreased to 1.8 IU/mL and anti-GBM titer decreased to 13.0 U/mL; however, his urine output did not recover. A CT scan ruled out a urological cause. As vasculitis-associated organ damage such as interstitial pneumonia, alveolar hemorrhage, or liver dysfunction was not observed from clinical findings and continued immunosuppressive treatment was thought to increase the risk of death from infection, the steroid dose was tapered off on the 43rd day of hospitalization. Although the dose of steroids was reduced, steroids could not be discontinued due to fever and a slight increase in MPO-ANCA titer. Subsequently, bacteremia, considered to be caused by Staphylococcus epidermidis identified by blood culture, occurred repeatedly and was treated with antibiotics while continuing hemodialysis. Catheter-associated infection and an abscess in the abdominal aorta were suspected, but no other localized sites of infection could be identified. On the 155th day of hospitalization, his C-reactive protein (CRP) levels increased again. Treatment with vancomycin and meropenem resulted in a poor response, and the patient died of septic shock on the 173rd day of hospitalization (Figure 2). After obtaining consent from the patient’s family, a pathological autopsy was performed.

**Figure 3:**
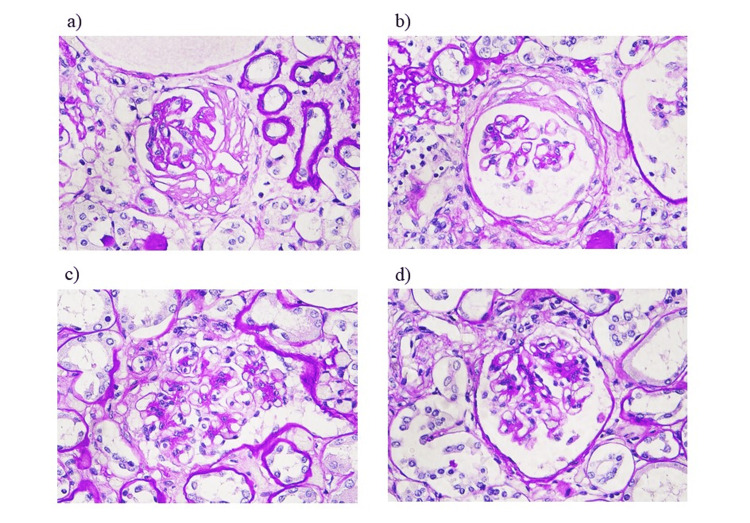
Renal pathological anatomical findings a) Fibrous crescent formation; b) Fibrocellular crescent formation; c) Endocapillary hypercellularity; d) Mesangial cell proliferation

**Figure 4:**
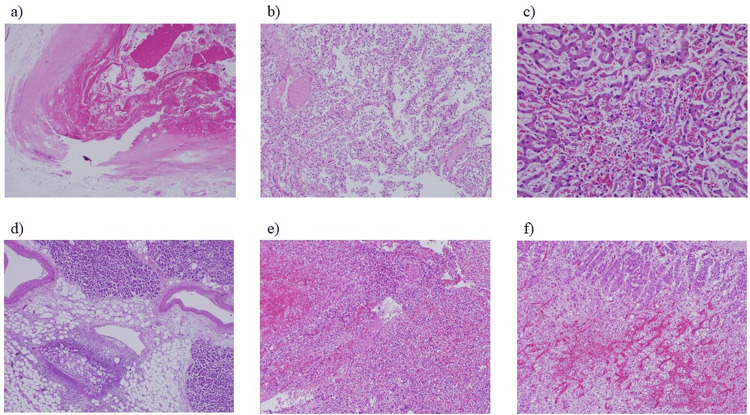
Extrarenal pathological findings a) In the abdominal aorta, bleeding, hematoma, and pseudoaneurysm formation are observed. These abdominal aortic findings were attributed to an abscess. b) In the alveoli, there is no evidence of inflammatory cell infiltration or vasculitis. c) In the spleen, there is centrilobular hepatocyte necrosis with congestion and hemorrhage. d) In the pancreas, there is fat necrosis due to sepsis and multiple organ failure. e) In the spleen, there is an infiltration of neutrophils. f) In the adrenal gland, there is a hemorrhage in the cortex.

Renal pathology revealed fibrocellular crescent formation in the glomeruli. Endocapillary hypercellularity and mesangial cell proliferation were also observed (Figure 3). Tubulointerstitium was severely involved with fibrocellular change and tubular atrophy in about 70% of the cortex. Immunofluorescence analysis revealed no significant deposition in the glomeruli. This result was suggestive of ANCA-associated nephritis. Pathological findings in the other organs did not suggest vasculitis (Figure 4). Gross pathological findings of the abdominal aorta showed that a part of the artificial blood vessel had formed a pseudoaneurysm and abscess, which were considered to have caused sepsis and multiple organ failure.

## Discussion

The patient in the present case was diagnosed with rapidly progressive glomerulonephritis, with positivity for three autoantibodies (MPO-ANCA, PR3-ANCA, and anti-GBM antibody). This case is valuable as such triple-positive cases are rare, and no autopsy reports have been presented.

Regarding the diagnosis in the present case, as the patient’s anti-GBM antibody titer was significantly elevated compared to the ANCA value, anti-GBM nephritis was initially assumed to be the cause; however, immunofluorescence analysis revealed no significant deposition in the glomeruli. Therefore, pathological findings suggested ANCA-associated vasculitis. Since this was a post-treatment, autopsy case, immunostaining of anti-GBM antibody IgG was reduced; therefore, it is possible that IgG deposition on the basement membrane was not observed. It could not be determined whether GBM nephritis was associated with this case. Anatomically, systemic vasculitis was rare and limited to the kidneys. Renal-limited vasculitis with elevated levels of multiple antibodies has been previously reported [1].

Anti-GBM antibodies have been reported to develop after ANCA [3]. Considering the clinical course and pathological findings of the present case, it was presumed that anti-GBM antibodies appeared after ANCA levels had increased. Therefore, it is reasonable to assume that ANCA-associated vasculitis was the predominant pathology. In a previous study analyzing the clinical characteristics of patients with both ANCA and anti-GBM antibodies, Jayne et al. reported that 30% of patients with anti-GBM antibodies had ANCA and 7.5% of those with ANCA had anti-GBM antibodies [4]. Hellmark et al. further reported that 38% of samples with anti-GBM antibodies had ANCA, and 14% of samples with ANCA had anti-GBM antibodies [5]. It is not clear why elevated ANCA led to elevated anti-GBM antibodies in the present case.

ANCA-associated nephritis is induced by environmental factors, systemic vasculitis is rare, and infections are believed to cause inflammation and damage to the glomerular basement membrane, exposing the target antigen and increasing GBM [3].

Regarding patient prognosis, no studies have yet compared triple-positive and non-positive patients. A study comparing the prognosis of ANCA alone, GBM alone, and ANCA + GBM reported that the renal prognosis was worse in anti-GBM antibody-positive patients than in those with ANCA alone [6]. Plasmapheresis has also been shown to be effective in treating ANCA-associated vasculitis and anti-GBM disease [7]. In the present case, plasma exchange therapy was administered immediately after starting steroid therapy. In addition, cyclophosphamide was administered to actively try to improve the renal prognosis. However, renal function did not recover, and renal replacement therapy was required. When treatment was started, the patient had already developed severe renal dysfunction with Cr levels of 7.16 mg/dL, and renal pathological recovery through treatment was considered difficult. It took 14 days from admission to the start of treatment, and it is speculated that renal function could not be recovered due to the delay in treatment. Treatment should have been started immediately after the diagnosis of RPGN.

In the present case, a renal biopsy was considered before treatment; however, this was not possible because one kidney was atrophied. Considering the patient's history of long-term hypertension and surgery for cardiovascular complications, the atrophy of one kidney was thought to be caused by a blood flow disturbance associated with arteriosclerosis. However, despite the risk, a renal biopsy should have been performed promptly for treatment.

Further, a pathological diagnosis was not possible prior to treatment, and the renal pathological findings were only confirmed after death, at which point pathological findings suggestive of localized vasculitis were observed in the kidney and not in other organs. When renal dysfunction was not expected to improve, immunosuppressive treatment should have been discontinued as soon as possible unless there was damage to other organs.

Initially, anti-GBM disease was suspected because of the markedly elevated GBM antibody titer; however, renal pathological immunostaining revealed a pauci-immune type; therefore, the possibility of anti-GBM disease was ruled out, and ANCA-associated nephritis was suggested.

According to clinical practice guidelines for rapidly progressive glomerulonephritis, anti-glomerular basement membrane nephritis can be diagnosed by hematuria or proteinuria and positive serum anti-GBM antibodies; therefore, a diagnosis can be made without pathological findings [7]. However, even if the diagnosis of anti-GBM disease is made based on clinical findings, the pathological findings may not agree, as in the present case. It was very unfortunate that this case was difficult to diagnose accurately during his lifetime and that he died of an infection during treatment. To accurately diagnose and understand the pathology of both ANCA-positive and GBM-positive cases, it is desirable to actively perform renal biopsies.

## Conclusions

We report a rare autopsy case of triple-seropositive RPGN. This case was attributed to ANCA-associated vasculitis, as renal pathology did not suggest the presence of anti-GBM disease. However, the correct diagnosis was unknown as no renal biopsy was performed prior to treatment. It was hypothesized that the increase in ANCA may have secondarily elevated the anti-GBM antibodies. If the renal biopsy had been performed early in this case, an improvement in prognosis may have been possible. In triple-seropositive RPGN cases, the assessment of pathology by renal biopsy is essential for proper diagnosis and treatment.
